# Performance of direct detection of *Mycobacterium tuberculosis* within *Mycobacterium tuberculosis* complex by routine MALDI-TOF for diagnosis using species-specific lipid fingerprint

**DOI:** 10.1128/spectrum.00356-25

**Published:** 2025-07-22

**Authors:** Bosco Cheong, Changchunzi He, Ian Laurenson, Pauline Claxton, Markus Kostrzewa, Francis Drobniewski, Gerald Larrouy-Maumus

**Affiliations:** 1 Department of Life Sciences, Faculty of Natural Sciences, Centre for Bacterial Resistance Biology, Imperial College London 98455, London, United Kingdom; 2 Adult Infectious Diseases and Center for Bacterial Resistance Biology, Department of Infectious Diseases, Imperial College London Department of Infectious Disease 170895, London, United Kingdom; 3 Scottish Mycobacteria Reference Laboratory, Edinburgh, United Kingdom; 4 Bruker Daltonics GmbH & Co. KG, Bremen, Germany; University of Heidelberg, Heidelberg, Germany

**Keywords:** MALDI, lipids, mycobacteria, identification

## Abstract

Managing tuberculosis cases requires species and drug susceptibility identification, which are limited by the time taken by testing procedures due to slow bacterial growth. Lipid-based matrix-assisted laser desorption/ionization-time-of-flight mass spectrometry (MALDI-TOF MS) is a promising tool for identifying pathogenic mycobacterial species. This study aims to define and use species-specific lipid profiles of members of the *Mycobacterium tuberculosis* complex (MTBC) obtained by MALDI-TOF MS, to directly discriminate *M. tuberculosis* from other members of the MTBC, such as *Mycobacterium africanum*, *Mycobacterium bovis*, and *M. bovis* bacillus Calmette-Guerin (BCG). Reference strains (*M. tuberculosis* H37Rv, *M. africanum*, *M. bovis*, and *M. bovis* BCG) were grown in Middlebrook 7H11 media (supplemented with 10% oleic acid-albumin-dextrose-catalase growth supplement) and incubated for up to 6 weeks at 37°C to generate the large biomass (~10^9^ bacteria) required for optimization, to assess reproducibility of the assay, and to set the reference lipid database. In clinical use, standard shorter culture periods would be sufficient. A blinded study was then performed using a collection of 46 mycobacterial clinical isolate strains composed of 30 *M*. *tuberculosis*, 2 *M*. *africanum*, 9 *M*. *bovis* BCG, and 5 *M*. *bovis* and grown under the same conditions. Cultured mycobacteria were heat-inactivated and loaded onto the matrix-assisted laser desorption target, followed by the addition of the matrix. Acquisition of the data was done using the negative ion mode. Using the species-specific glycolipid, sulfolipids, *M. tuberculosis* was discriminated within MTBC using the MALDI-TOF process with a sensitivity and specificity of 86.7% (95% confidence interval [CI] 69.3–96.2) and 93.7% (95% CI 69.8–99.8), respectively. Direct detection of *M. tuberculosis* within the MTBC based on mycobacterial lipid profiling provides a safe and accurate method, based on the detection of the sulfolipids, as a species-specific lipid biomarker of *M. tuberculosis*.

**IMPORTANCE:**

Tuberculosis remains a major infectious disease in humans and mammals, but one of the major challenges is to accurately discriminate *M. tuberculosis* from the other mycobacterial species belonging to the MTBC. Here we report on a novel assay that can detect *M. tuberculosis* directly within the MTBC. This approach is simple and relies on the detection of species-specific lipids by routine MALDI-TOF MS.

## INTRODUCTION

The *Mycobacterium tuberculosis* complex (MTBC) is a group of closely related species of mycobacteria that causes tuberculosis (TB) in humans and animals; members include *Mycobacterium tuberculosis* (Mtb), *Mycobacterium bovis*, *Mycobacterium bovis* bacillus Calmette-Guerin (BCG), *Mycobacterium africanum*, *Mycobacterium canettii*, *Mycobacterium microti*, *Mycobacterium caprae*, and *Mycobacterium pinnipedii*, among others (1–4). Prior to the coronavirus disease 2019 (COVID-19) pandemic in 2020, although the global mortality rate was projected to decline, TB was estimated to be the 13th leading cause of death worldwide (5). Since the COVID-19 pandemic ended, the TB mortality rate has reverted to an increasing trajectory and remains one of the two greatest causes of death from a single infectious agent (5). This was believed to have resulted from diverting significant resources and attention to remedy the pandemic, inhibiting efforts in TB diagnosis and treatment (5–7).

Most cases of TB infections in humans are caused by *M. tuberculosis*. Different MTBC members are associated with different degrees of virulence, disease severity, and inherent drug susceptibility (8); hence, it is clinically important to identify pathogenic mycobacteria at the species level. Conventional methods of microbial identification include a range of microscopic, biochemical, and molecular tests, which have unique advantages and disadvantages (9). In general, the drawbacks of most available methods with respect to their use in routine clinical diagnosis are that they are costly and have below-desirable reproducibility. In the case of TB, MTBC members are over 99% genetically similar and possess an identical 16S rRNA gene (10), where differentiation by nucleotide-based methods is not routinely possible. These disadvantages limit accessibility and how early preventative measures and treatments can be put in place, subsequently affecting disease transmission, morbidity, and potential mortality rates. With MTBC identification complicated by additional persistent and, in some countries, rising multidrug and rifampicin-resistant phenotypes in TB cases (5), accurately identifying MTBC species and drug resistance is imperative to the global management of TB.

The current approach to TB diagnosis recommended by the World Health Organization, including species identification and drug susceptibility, utilizes a combination of rapid molecular assays, microscopy and microbiological culture, and whole or targeted genome sequencing or phenotypic susceptibility testing (5). Bacteriological culture-based methods of diagnosis are not always accessible. The median proportion of bacteriologically diagnosed pulmonary TB cases in 2022 was nearly 20% lower in low-income countries than in high-income countries (5). Due to the high costs of bacteriological diagnoses, lower-income countries rely on lower-cost rapid TB tests such as sputum smear microscopy and lateral flow urine lipoarabinomannan assays in key groups, which are less sensitive (11, 12). This disparity in diagnosis further highlights the need for developing more rapid, cost-effective TB diagnostic tools accurately identifying species to ensure the most appropriate treatment is administered and to help trace sources of infection caused by disease transmission.

Matrix-assisted laser desorption/ionization-time-of-flight mass spectrometry (MALDI-TOF MS) is an emerging technique that has revolutionized clinical microbiology (13–15). Compared to conventional means of TB diagnosis, MALDI-TOF MS is simpler to use, of lower long-term cost and turnaround time for analysis, and available in thousands of laboratories over the world. Only positive ion mode-based protein/peptide analysis was available for commercially accessible MALDI-TOF MS platforms until recently, so protein-based MALDI-TOF MS has been the gold standard in bacterial identification, in which in the case of mycobacteria, MALDI-TOF MS could be run at around 2 weeks upon primary mycobacterial growth of colonies. However, this method is not simple for identifying microbes with thick outer cell walls, and sometimes identification is limited to a group/complex level. For *Mycobacteria* sp., the mycobacterial cell wall has a rich diversity of lipids, including species-specific lipids. However, unique components such as mycolic acid render the cell wall extremely apolar; therefore, laborious methods of cell lysis and protein extraction must be performed as part of the sample preparation (16–21). Additionally, *Mycobacterium* subspecies-level differentiation within MTBC has not been achieved by protein-based MALDI-TOF MS (22–24). Once negative ion mode methodology became prevalent, studies have increasingly focused on analyzing lipid profiles from negative ion mode and positive ion mode for protein-based bacterial identification.

We recently proposed a novel approach that aims to identify *M. tuberculosis* and non-tuberculous mycobacteria and *M. tuberculosis* within the MTBC based on MALDI-TOF MS analysis of lipids from heat-inactivated bacteria (25–29). With the unique richness and composition of lipids making up the mycobacterial cell wall (17, 18, 30, 31), the cell envelope becomes a reservoir of potential diagnostic biomarkers. Among these is found sulfolipid-I (SL-I), a cell wall lipid exclusive to *M. tuberculosis* and a virulence factor believed to contribute to pathogenicity in several ways (32–37). These species-specific lipids have the potential to be used as biomarkers for *M. tuberculosis* identification and for discriminating members within the MTBC. The aim of this study was to test and establish a methodology to differentiate *M. tuberculosis* and key subspecies within the MTBC using SL-I lipid profiling with a commercially available MALDI-TOF MS system.

## MATERIALS AND METHODS

### Clinical *Mycobacterium tuberculosis* complex isolate preparation and study design

A total of 46 MTBC clinical isolates from the Scottish Mycobacteria Reference Laboratory (SMRL) were cultured from frozen aliquots onto Middlebrook 7H11 broth supplemented with oleic acid-albumin-dextrose-catalase without glycerol (Sigma-Aldrich Company Ltd., Gillingham, Dorset, UK) and incubated for 3–6 weeks at 37°C. The isolates comprised 30 *M*. *tuberculosis*, 2 *M*. *africanum*, 9 *M*. *bovis* BCG, and 5 *M*. *bovis*. Four reference strains were used to produce a database of profiles using cultures of *M. tuberculosis* H37Rv, *M. africanum*, *M. bovis*, and *M. bovis* BCG.

An algorithm for analysis was prepared based on the reference culture profiles and literature and then applied to the spectra obtained in a validation cohort of 46 MTBC strains (File S3).

Clinical MTBC strains were identified by the SMRL using a combination of microscopy and commercial molecular line probe assays available from Bruker (Hain Lifescience, a Bruker Microbiology company, Nehren, Germany) and whole-genome sequencing (38, 39). MTBC cultures were selected from the SMRL collection independently and grown to an appropriate density and recoded before being transferred to a third laboratory for the matrix-assisted laser desorption (MALDI) analysis.

### Sample preparation

Once the optimal growth of bacteria was obtained (typically 3–4 weeks for 10^7^ bacteria and 4–6 weeks for 10^9^ bacteria), 100 µL of bacterial suspension was placed into 1.5 mL Eppendorf tubes and heat-killed at 95°C for 30 minutes before leaving the biosafety level 3 containment area. The heat-killed MTBC pellets were washed four times with 200 µL of double-distilled water using a microfuge set at 15,000 × *g* for 2 minutes at room temperature. Fifteen microliter aliquots were transferred from each of the 46 MTBC clinical isolates into 1.5 mL microtubes. The aliquots were heat-dried at 98°C for 10 minutes to make a film at the bottom of the 1.5 mL microtubes. Three microliters of matrix was mixed with each dried pellet by pipetting up and down; 0.5 µL of the mixtures was pipetted onto the MSP 96 target polished steel BC (Bruker Daltonics, Germany; #8280800). The matrix used consisted of the Lipid Xtract Matrix from the MBT Lipid Xtract Kit (Bruker Daltonics) mixed with 75 µL of 100% isopropanol per vial. The components are easily commercially available. All solvent manipulations and handling were carried out under a fume hood.

### Mass spectrometry analysis

MS analyses were performed on a MALDI Biotyper sirius System (Bruker Daltonics). The mass spectra were scanned in the range of *m*/*z* 1,000–3,000. The mass profiles were acquired using FlexControl 3.4 software (Bruker Daltonics). The spectra were recorded in the linear negative-ion mode (laser intensity 65%, ion source 1 = 15.00 kV, ion source 2 = 13.70 kV, lens = 4.45 kV, detector voltage = 2698 kV, and pulsed ion extraction = 200 ns). Each spectrum corresponded to ion accumulation of 2,000–5,000 laser shots randomly distributed on the spot. The spectra obtained were processed with default parameters using FlexAnalysis v.3.4 software (Bruker Daltonics).

### Statistical analysis

An initial database of lipidomic profiles was created from the culture of four reference MTBC strains (*M. tuberculosis* H37Rv, *M. africanum*, *M. bovis*, and *M. bovis* BCG). Subsequently, 46 MTBC clinical isolate cultures were regrown to an optimum density from frozen stocks. All 46 cultures were then coded and submitted for blinded MALDI analysis. Analysis of the mass spectra of the clinical isolates was conducted by randomly recoding the cultures from the SMRL and utilizing two operators who independently identified the cultures using the MALDI lipidomic spectra and were blinded to the original reference culture results. Experiments were performed as two technical replicates. Sensitivity, specificity, and 95% confidence interval (CI) were calculated as described in the literature (40–43).

## RESULTS

**Fig 1:**
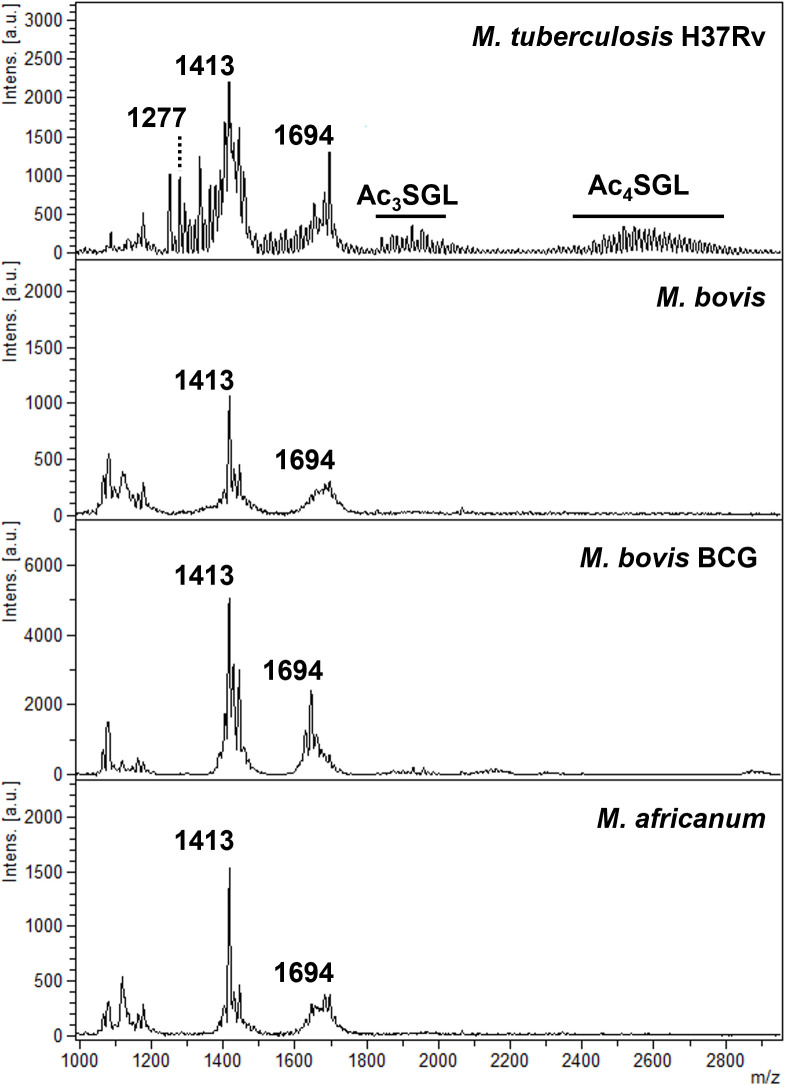
Mass spectra of mycobacteria belonging to the *M. tuberculosis* complex. Mass spectra were acquired in the negative ion mode using the MBT lipid xtract matrix as matrix solubilized in 2-isopropanol.

The study examined first the MTBC lipidomic profile by MALDI-TOF mass spectrometry in the negative ion mode for the reference strains to identify unique markers. From the MALDI lipid profiles generated from the reference strains (Fig. 1) and based on the literature, we propose the following analysis and interpretation. In the negative ion mode, the *M. tuberculosis*, *M. bovis*, *M. bovis* BCG, and *M. africanum* spectra are dominated by a set of peaks at *m*/*z* 1,413 assigned to *m*/*z* 1,694 assigned to phosphatidyl-*myo*-inositol-dimannosides containing three fatty acids (C16:0, C16:0, and C19:0) and four fatty acids (C16:0, C16:0, C19:0, and C19:0), respectively. In addition, only in the *M. tuberculosis* strain, the mass range shows a set of peaks from *m*/*z* 1,250 to 1,300, from *m*/*z* 1,800 to 2,000, and from *m*/*z* 2,400 to 2,800 attributed to diacylated, triacylated, and tetra-acylated sulfolipids, respectively. Indeed, *m*/*z* 1,000 and 3,000 are dominated by a set of pseudomolecular ions distant of 14 mass units from *m*/*z* 1,250 to 1,300, from *m*/*z* 1,800 to 2,000, and from *m*/*z* 2,200 to 3,000 (Fig. 1; Files S1 and S2), typifying the latest tetra-acylated sulfoglycolipids (Ac4SGL) with three hydroxyphthioceranoic acids and one C16 or C18 fatty acid residues. Sulfoglycolipids were first described by Goren et al. as “sulfatides” (33–35, 37) and were found only in the envelope of *M. tuberculosis* strains but not in *M. bovis* BCG, *M. bovis*, nor *M. africanum* strains belonging to the *M. tuberculosis* complex. Those profiles obtained from the reference strains were then used to determine the performance of the signatures found against a collection of 46 MTBC clinical isolates provided by the SMRL.

The mass spectra of the 46 MTBC isolates were acquired in negative ion mode, which is the ion mode suitable to detect SL-I. MALDI interpretations were then made blind to these results.

For the 46 clinical isolates, a good mycobacterial mass spectral signal is defined by a resolution greater than 100 and a signal-to-noise greater than 3 in the negative ion mode for the signature peaks identified from the reference strains cited earlier. Out of 46 spectra generated corresponding to the 46 MTBC clinical isolates, 42 spectra matched the desired criteria (resolution >100 and signal-to-noise >3) and were interpretable. The four isolates that could not be assigned to either the Mtb or non-Mtb species group could be caused by poor crystallization on the MALDI target plate, possibly due to contamination by non-ionic detergent arising from the culture medium. Out of 42 isolates that generated good MALDI spectra and were interpretable, 41 were correctly assigned (Table 1), based on the negative mode signature mentioned above.

**TABLE 1:** Sensitivity and specificity of the lipid fingerprint-based MALDI-TOF method

***M. tuberculosis*** identification (*n* = 46)		Whole-genome sequencing/molecular line probe assay identification			
		Positive	Negative	Sensitivity (%)	Specificity (%)
MALDI-TOF	Positive	26	1	86.7 (95% CI 69.3–96.2)	93.8 (95% CI 69.8–99.8)
	Negative	4	15		

The sensitivity and specificity of the MALDI-TOF were 86.7% (95% CI 69.3–96.2) and 93.7% (95% CI 69.8–99.8), respectively (Table 1).

## DISCUSSION

The lipidomics approach, using the identification of surface-exposed species-specific lipids, can discriminate Mtb within MTBC, unlike current proteomic approaches (44–48). It is performed in a straightforward two-step process and can discriminate *M. tuberculosis* directly from *M. bovis* BCG, *M. bovis*, and *M. africanum*.

MTBC cultures rendered safe by heat inactivation generated a high-quality interpretable spectrum in 91% (42 out of 46) of samples by MALDI-TOF MS using an intact bacterial lipidomics approach; 4 out of 46 samples did not generate sufficient quality spectra for analysis.

The sensitivity and specificity for isolates for which a spectrum was identifiable indicate that the novel method is a very promising tool for the identification of mycobacteria by the routine application of the MALDI-TOF. MBT sirius is an example of the next generation of clinical microbiology MALDI-TOF systems operating both in positive and negative ion mode.

### Limitations of study

We could not differentiate *M. bovis* BCG from *M.* bovis. Additionally, this study does not contain clinical *M. canettii* isolates due to the extremely rare infections caused by this subspecies in the UK and globally. It has also been argued that *M. canettii* is not properly a member of the MTBC but should be considered as a closely related but separate clade of tubercle bacilli (49, 50).

In addition to future medical diagnostic use, the methodology, after further development, validation, and registration, is likely to be of value to the veterinary sector; i.e., proof of animal tuberculosis is critical, especially if disease can be correctly distinguished from vaccination (51–53).

As part of this process, in combination with the existing mycobacteria module (Bruker Daltonics), which has limited application to discriminate mycobacteria within the MTBC, the lipid fingerprint approach could be integrated as part of this module to increase the specificity of the test beyond the complex level, allowing rapid differentiation and simplifying existing workflows for discrimination of *M. tuberculosis* within the MTBC.

## Data Availability

Raw data are found in the supplemental material.

## SUPPLEMENTAL MATERIAL

File S1 spectrum.00356-25-s0001.xlsx Raw data for MTB strains.

File S2 spectrum.00356-25-s0002.xlsx Raw data for non-MTB strains.

File S3 spectrum.00356-25-s0003.xlsx List of clinical isolates used in this study and their classification based on the MALDI-ToF spectra obtained in the negative ion mode.

ASM does not own the copyrights to Supplemental Material that may be linked to, or accessed through, an article. The authors have granted ASM a non-exclusive, world-wide license to publish the Supplemental Material files. Please contact the corresponding author directly for reuse.
